# Uterus Dysplasia Associated with Cervico-Vaginal Agenesis

**DOI:** 10.22074/ijfs.2018.5111

**Published:** 2017-10-14

**Authors:** Ali Mahdavi, Hadi Mirfazaelian, Ladan Younesi asl, Zeynab Hasani, Maryam Bahreini

**Affiliations:** 1 Department of Radiology, Tehran University of Medical Sciences, Tehran, Iran; 2 Department of Emergency Medicine, Tehran University of Medical Sciences, Tehran, Iran; 3 Department of Radiology, Iran University of Medical Sciences, Tehran, Iran Abstract

**Keywords:** Amenorrhea, Magnetic Resonance Imaging, Müllerian Duct Hypoplasia

## Abstract

Müllerian ducts can form upper parts of normal female reproductive system and any failure in ductal fusion may result in to müllerian duct anomalies (MDA). We present a case of MDA and a uterus dysplasia with no evidence of cervical or upper vaginal tissue. This case showes the role of magnetic resonace imaging (MRI) on MDA diagnosis and urges the need for a unified reliable and practical classification more compatible with clinical practice.

## Introduction

Recent studies have challenged the embryological origin of female genitourinary tract especially vagina as some rare types of anomalies were reported. According to one theory, the embryological development of the vagina proceeds from the mesonephric ( Wolffian ) ducts and the Müllerian tubercle instead of the Müllerian ducts and the urogenital sinus (1). Several Genetic mutations may lead to müllerian duct anomalies ( MDAs ). The most common classification system for MDAs was developed by the American Fertility Society ( AFS ) (2). It is important to know that this classification system is only a framework and that not all anomalies will fit completely into one of the categories (1). We present a patient with uterus dysplasia related to cervical and incomplete vaginal agenesis which is classified variably according to the present classifications. Informed consent was obtained from the adult participant. The Ethics of this report have been approved by Tehran University of Medical Sciences Institutional Review Board. 

## Case report

**Fig.1:**
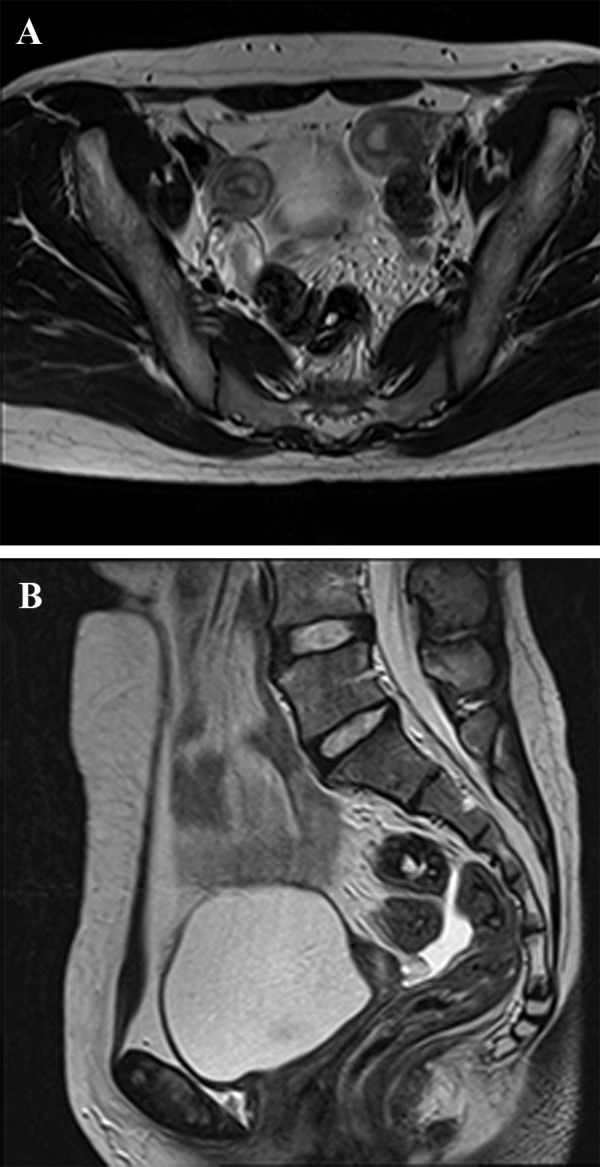
Magnetic resonance imaging study of the patient with cervico-vaginal agenesis. A. Axial T2 Weighted image shows two separated uterus with normal endometrial cavity and B. In Sagittal T2 Weighted image, cervix and 1/3 of upper vagina is not visible. Mild to moderate fluid is also present within pelvic cavity.

An 18-year-old girl referred to our institute for primary amenorrhea evaluation. She had normal growth pattern with no sign of periodic pelvic pain, pelvic discharge, or bleeding. The past history was negative for diethylstilbestrol exposure in her mother and no case of MDA was present in her first relatives. On a physical examination, her external genitalia was normal, she had hymen and permeable vagina and her secondary sexual characteristics ( including breast budding and axillary and groin hair ) appeared to be normal. The patient had incomplete vaginal agenesis: lack of vagina in the upper one-third with normal lower two-thirds. There were no signs of abdominal and pelvic mass, previous surgical scar, or any urological abnormality. Laboratory studies were within normal limits. She underwent a trans-abdominal ultrasound examination revealing separate divergent uterine horns with separate endometrial cavities, normal ovaries bilaterally and no evidence of cervical or upper vaginal tissue. Consequently, MRI was obtained for further evaluation. (Fig .1A,B). On the MRI, two small uteri were noted in the pelvic fossa with normal endometrium within them in axial T2 images without contrast administration (Fig .1A). In sagittal T2 images, cervix and one-third of upper vagina were not visible (Fig .1B). Mild to moderate fluid appeared within pelvic cavity but no signs of hydrometrocolpos or hematometrocolpus were seen. There was also no evidence of renal agenesis, abnormalities of ureters or skeleton. The patient was diagnosed with a MDA referred to gynecologist for treatment. 

## Discussion

In this study, we have presented a case of uterus dysplasia with cervical and incomplete vaginal hypoplasia. MDAs are from the cause of interruption or dysregulation in Müllerian duct development at various stages of morphogenesis. Multiple etiologies such as genetic factors and teratogenic drugs ( e.g. diethylstilbestrol, thalidomide ) are related to these anomalies. The genetics of MDA are complex and not fully understood. Generally, they occurred sporadically but other modes of inheritance, such as autosomal dominant, autosomal recessive, and X-linked disorders have been reported. Also, it could be a component of a complex malformation syndrome (3). Uterus dysplasia is associated with premature birth, malpresentation, intrauterine growth retardation, spontaneous abortion and infertility. 

In 1988, the AFS classified MDA into 7 major categories (2). Based on this method of classification, failure to go through developmental stages leads to a specific malformation. However, observed anomalies are not mutually exclusive and many MDAs often coexist. Also several cases of MDAs have been reported which fail to be classified as one of AFS classification categories ( such as normal uterus with double cervix and vagina (4), septate uterus with cervical duplication (5), bicervical uterus with and without septate vagina (6), or double cervix and vagina with septate uterus (7). According to the European Society of Human Reproduction and Embryology ( ESHRE ) consensus, uterine anatomical abnormalities are described as following: U0, normal uterus; U1, dysmorphic uterus; U2, septate uterus; U3, bicorporeal uterus; U4, hemi-uterus; U5, aplastic uterus; U6, unclassified cases and cervical and vaginal anomalies are added subsequently. Our patient was in class I of the AFS and a U5a/Cervix4/Vagina4 ESHRE classification, group 3A of complex female genital malformations, IIIA1C of the embryological and clinical classification (2,8,11). Applying MRI as the correct modality of choice for assessing of MDAs and sonohysterography are helpful to noninvasively examine the uterine anomalies (12). 

## Conclusion

Our observations together with recent discoveries in pathogenesis and genetics of MDAs may affect future investigations on more clinically practical classifications of MDAs. Indeed, more research projects on pathophysiological and genetic aspects of these anomalies are required for unifying the classification of MDAs. 
